# MALDI-TOF Mass Spectrometry Analysis and Human Post-Mortem Microbial Community: A Pilot Study

**DOI:** 10.3390/ijerph19074354

**Published:** 2022-04-05

**Authors:** Carlo Pietro Campobasso, Gennaro Mastroianni, Alessandro Feola, Pasquale Mascolo, Anna Carfora, Bruno Liguori, Pierluca Zangani, Federica Dell’Annunziata, Veronica Folliero, Arianna Petrillo, Maria Elena Della Pepa, Francesca Martora, Marilena Galdiero

**Affiliations:** 1 Department of Experimental Medicine, Legal Medicine Section, University of Study of Campania “Luigi Vanvitelli”, 80138 Naples, Italy; carlopietro.campobasso@unicampania.it (C.P.C.); rinomastroianni@gmail.com (G.M.); mascolopasquale2@gmail.com (P.M.); anna.carfora@unicampania.it (A.C.); bruno.liguori@gmail.it (B.L.); pierluca.zangani@unicampania.it (P.Z.); 2 Department of Experimental Medicine, Microbiology Section, University of Study of Campania “Luigi Vanvitelli”, 80138 Naples, Italy; federica.dellannunziata@unicampania.it (F.D.); veronica.folliero@unicampania.it (V.F.); mariaelena.dellapepa@gmail.com (M.E.D.P.); francescamartora@gmail.com (F.M.); marilena.galdiero@unicampania.it (M.G.); 3 Pediatric Unit, Fondazione IRCCS “Ca’ Granda-Ospedale Maggiore-Policlinico”, 20122 Milan, Italy; arianna.petrillo30@gmail.com

**Keywords:** human post-mortem microbiome, MALDI-TOF, post-mortem interval, thanatomicrobiota, epinecrotic communities

## Abstract

Introduction: The human post-mortem microbiome (HPM) plays a major role in the decomposition process. Successional changes in post-mortem bacterial communities have been recently demonstrated using high throughput metagenomic sequencing techniques, showing great potential as a post-mortem interval (PMI) predictor. The aim of this study is to verify the application of the mass spectrometry technique, better known as MALDI-TOF MS (matrix-assisted laser desorption/ionization time-of-flight mass spectrometry), as a cheap and quick method for microbe taxonomic identification and for studying the PM microbiome. Methods: The study was carried out on 18 human bodies, ranging from 4 months to 82 years old and with a PMI range from 24 h up to 15 days. The storage time interval in the coolers was included in the final PMI estimates. Using the PMI, the sample study was divided into three main groups: seven cases with a PMI < 72 h; six cases with a PMI of 72–168 h and five cases with a PMI > 168 h. For each body, microbiological swabs were sampled from five external anatomical sites (eyes, ears, nose, mouth, and rectum) and four internal organs (brain, spleen, liver, and heart). Results: The HPM became increasingly different from the starting communities over time in the internal organs as well as at skin sites; the HPM microbiome was mostly dominated by Firmicutes and Proteobacteria phyla; and a PM microbial turnover existed during decomposition, evolving with the PMI. Conclusions: MALDI-TOF is a promising method for PMI estimation, given its sample handling, good reproducibility, and high speed and throughput. Although several intrinsic and extrinsic factors can affect the structure of the HPM, MALDI-TOF can detect the overall microbial community turnover of most prevalent phyla during decomposition. Limitations are mainly related to its sensitivity due to the culture-dependent method and bias in the identification of new isolates.

## 1. Introduction

Bacteria play a crucial role in human health and decomposition [1,2,3]. Recent advances in microbiology and metagenomic technologies have demonstrated that components of the human post-mortem microbiome (HPM) include bacterial communities of the internal organs (also called thanatomicrobiota) and of superficial epithelial and mucosal tissues (also called epinecrotic) [4,5,6]. The advent of advanced sequencing techniques has made it feasible to provide fine resolution of microbial taxonomic characterization (e.g., family, genus, and species), which was not always possible with traditional and culture-based methods [7,8]. Successional changes in post-mortem (PM) bacterial communities have been demonstrated using high throughput metagenomic techniques with potential application for the determination of the post-mortem interval (PMI) [9,10,11]. However, the microbiota of living hosts is highly influenced by the environment [12,13] and is variable from person to person and between different body sites in a single host according to age and sex [14,15,16], presence or absence of disease, illicit or prescribed drugs, and nutrition [17]. Aim of this study is to verify the potential of mass spectrometry as a cheap and quick method for microbe taxonomic identification by studying the variability over decomposition and among anatomical sites of the epinecrotic bacteria communities as well as of the thanatomicrobiota. Matrix-assisted laser desorption/ionization time-of-flight mass spectrometry (MALDI-TOF MS) has been recently proposed in forensic settings for estimating the PMI in animal models (rats) because of its capability to obtain molecular images and data relating to many endogenous and exogenous molecules without the need for target-specific reagents [9,18,19,20]. MALDI-TOF MS is becoming commonly applied for molecular diagnosis and classification of tumors [21,22] and also for microbial identification and diagnosis of a broad spectrum of microbes ranging from Gram positive to Gram negative bacteria [23,24]. The identity of a microorganism can be established down to the genus, and, in many cases, to the species and strain level [25]. Although currently microorganisms are best identified using biochemical analyses and 16S or 18S rRNA gene sequencing, MALDI-TOF has expanded significantly over recent years and is widely utilized as a rapid and sensitive method for the identification of microorganisms in a variety of fields, such as clinical diagnosis, food monitoring, ecological research, and military science [26,27,28,29]. MALDI-TOF MS has been also recommended as robust method, economical in terms of both labor and costs involved, because of minimal requirements for substrates and reagents for extraction [30,31]. In fact, microbial identification using MALDI TOF MS is based on identifying a characteristic spectrum of each species, which is then matched with a large database within the instrument. The characteristic spectrum is also known as the peptide mass fingerprint (PMF) that is generated for the analytes in the unknown sample to be matched with the known PMF [24,27,30]. The limitation of the technology is that identification of new isolates is possible only if the spectral database contains a PMF of the type strains of specific genera/species/subspecies [24]. Based on this knowledge, this is a pilot study on the potential of the MALDI-TOF MS platform to provide information on the epinecrotic and internal bacteria communities according to the PMI and anatomical site.

## 2. Materials and Methods

The study was carried out on 18 human bodies stored temporarily in the mortuary before a forensic autopsy. The sample group represented 17 males and just one female, with age range from 4 months to 82 years old (mean age of 43.27 years) and a PMI range from 24 h up to 15 days. In most of the cases (15 out of the total 18), death was witnessed as it occurred in domestic or clinical settings. Therefore, each PMI was also determined based on circumstantial information about the lethal event as well as eyewitness reports. The interval period between death and the placement of the body in the mortuary was just a few hours after death for all bodies found indoors and outdoors. After 24–36 h in the mortuary, all bodies were kept refrigerated at +4 °C before the autopsy that was performed in most of the cases (10 out of the total 18) within 72–96 h after death. In order to avoid contamination, the bodies were kept in single body cabinets so that there was no simultaneous presence of bodies inside the same refrigerator. The single body cabinets were sterilized between the storage of one corpse and the next. Unfortunately, for some of the bodies, the autopsy was only performed after several days for administrative and judicial reasons. For these reasons, it was not possible to take microbiological samples at several regular intervals because the bodies were of judicial interest. The storage time interval in the mortuary and in the coolers was included in the final PMI. Therefore, according to the PMI, the 18 cases were divided into 3 main study groups: 7 cases with a PMI of <72 h; 6 cases with a PMI of 72–168 h, and 5 cases with a PMI of >168 h.

### 2.1. Autopsy and Sampling

For each body, at the beginning of the autopsy microbiological swabs were sampled from five external anatomical sites (eyes, ears, nose, mouth, and rectum) and four internal organs (brain, spleen, liver, and heart). For each location, an individual swab was rubbed while being rotated for few a seconds to thoroughly sample the associated microbial community. In order to avoid contamination, all instruments used for the internal section of the body (i.e., manual and oscillating autopsy saws, sharp blades, scissors, etc.) were sterilized between the autopsy of one corpse and the next. 

### 2.2. Microbiological Analysis

After sampling, swabs were transferred for culturing in the microbiological laboratory, to characterize the culturable aerobic and anaerobic bacteria communities and also yeast, associated with dead bodies. Samples were then streaked in four different agarized plates as follows: Columbia CNA agar with 5% Sheep Blood, MacConkey agar, Chocolate agar, and Sabouraud agar (Biomerieux, Marcy l’Etoule, France). The media plates were incubated aerobically at 37 °C for 24 h, except for the Sabouraud agar plates which were incubated at 30 °C for 48 hours. In addition, the Chocolate agar was incubated in anaerobiosis conditions. 

### 2.3. Bacterial Identification

The cultured plates were examined, and the bacterial and yeast isolates were identified and characterized using MALDI-TOF (Bruker Daltonics, Bremen, Germany) [32]. For MALDI-TOF analysis, an isolated single colony from culture plates was spotted onto a 96 metallic target plate, air dried and then overlaid with 1 μL of a α-cyano-4 hydroxycinnamic acid (HCCA) matrix solution. The sample matrix was left at room temperature and then introduced into the mass spectrometer for data acquisition. The sample spots were shot using laser desorption/ionization and mass spectra, represented by mass to change ratios (m/z), were obtained. Software automatically generated the MALDI-TOF spectrum for each microorganism, also called the peptide mass fingerprint (PFM). The PFM spectrum of unknown organisms was then matched with the proteome database reference to provide microbial identification. For each isolate, mass spectra were obtained using Flex Control Software (Bruker Daltonics, Bremen, Germany).

## 3. Results

The details of each case (sex, age, death scene, PMI, stage of decay, manner, and cause of death) are summarized in Table 1.

Cause and manner of death were assessed only at the end of the autopsy according to the results of ancillary investigations (i.e., toxicological and histological analyses). The distribution of the cause and manner of death along the study group shows 9 natural deaths (2 cardiac deaths due to myocardial infarction and arrhythmogenic cardiomyopathy, and 3 respiratory deaths due to pneumonia) and 9 violent deaths (3 suicides, 3 car accidents, and 3 homicides by blunt and ballistic injuries). Violent and natural deaths were considered eligible for the study in order to investigate the potential of MALDI-TOF to detect the overall bacteria dynamics of the epinecrotic community and thanatomicrobiota according to PMI and anatomical site. 

**Figure 1:**
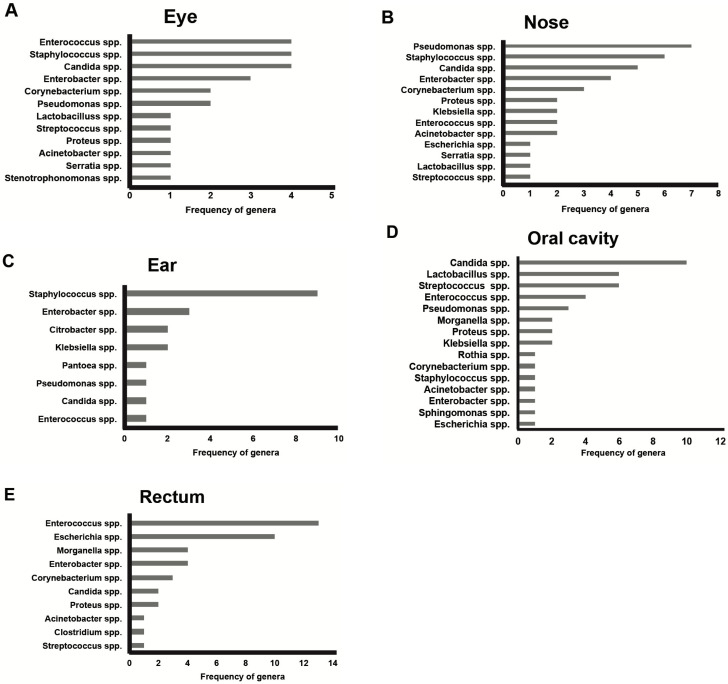
The distribution of genera among the five external anatomical sites: (**A**) eye; (**B**) nose; (**C**) ear; (**D**) oral cavity and (**E**) rectum.

**Figure 2:**
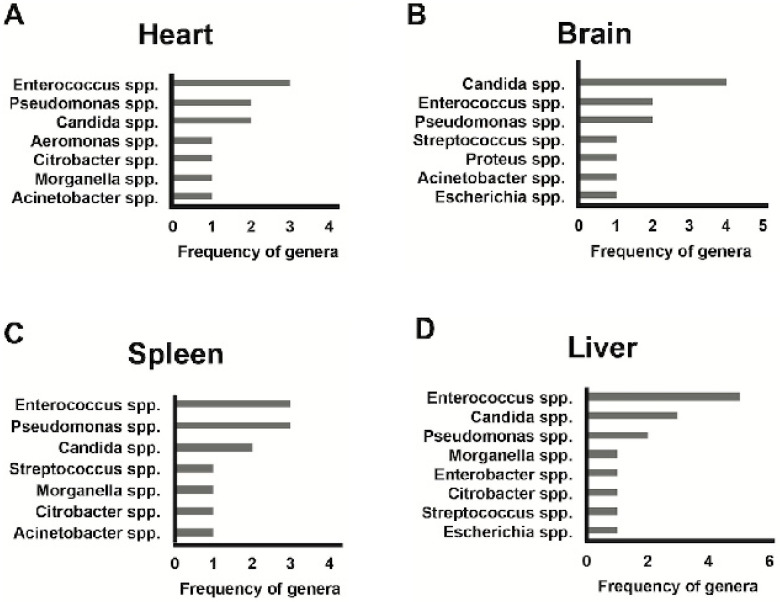
The distribution of genera among the four internal anatomical sites: (**A**) heart; (**B**) brain; (**C**) spleen and (**D**) liver.

**Figure 3:**
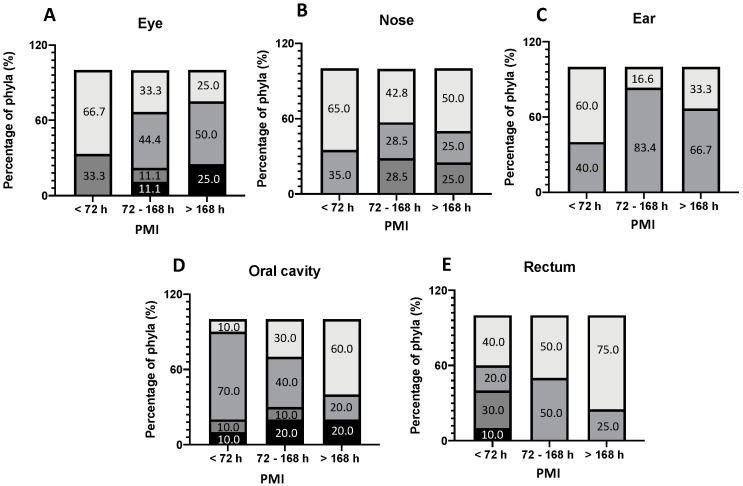
The frequency of phyla among the five external anatomical sites according to PMI. (**A**) eye; (**B**) nose; (**C**) ear; (**D**) oral cavity and (**E**) rectum. [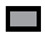 Firmicutes, 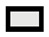 Proteobacteria, 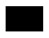 Ascomycota, 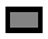 Actinobacteria].

**Figure 4:**
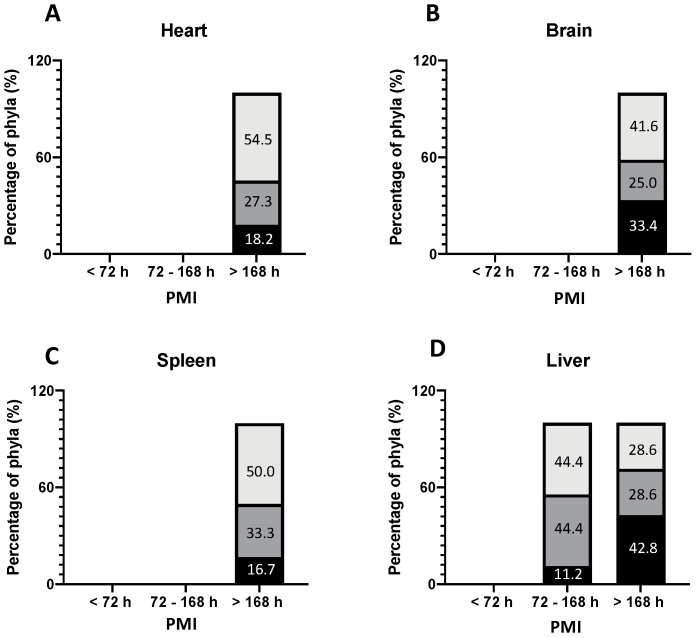
The frequency of phyla among the four internal anatomical sites according to PMI. (**A**) heart; (**B**) brain; (**C**) spleen and (**D**) liver. [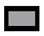 Firmicutes, 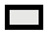 Proteobacteria, 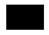 Ascomycota, 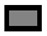 Actinobacteria].

For most of the bodies (15 out of 18), death occurred in enclosed environments, as follows: 10 cases in hospitals, 4 cases in apartments, and one case in jail. Only three bodies were found outdoors, including one suicide (a fatal fall from great height) and 2 natural deaths. For these bodies, no signs of insect activity were observed. At the autopsy, most of the bodies (13 out of the total 18) were well-preserved and largely fresh in accordance with their early PMI of <168 h, including the period spent in the coolers after death. Only four bodies out of six cases with a PMI of between 72 and 168 h showed a few signs of early decomposition such as green discoloration at the iliac right fossa and some marbling appearance at the upper thorax or upper limbs. Signs of more advanced decomposition, such as initial bloating of the scrotum and purging of decomposition fluids were observed in only some of the five bodies with a PMI of >168 h, including one victim who died due to septic shock. The results of microbiological analyses are summarized in Figure 1, Figure 2, Figure 3 and Figure 4. Only 10 swabs out of a total of 162 taken from the 18 cases were sterile and they only came from the eye (five samples) and from the ear (five samples). The bacteria cultured from the epinecrotic and thanatomicrobiota areas were grouped in phyla, genera, families and species. In particular, from the 18 corpses, bacterial communities represented 4 main phyla, 13 families, 20 genera, and 45 species. All data are available as supplementary electronic material. For the epinecrotic community, the distribution of genera found among the five external anatomical sites (eyes, ears, nose, mouth, and rectum) is shown in Figure 1.

For the thanatomicrobiota, the distribution of genera found among the four internal organs (brain, spleen, liver, and heart) is depicted in Figure 2. The most common genera found in the eye were *Enterococcus* spp., *Staphylococcus* spp., and *Candida* spp. These genera were also prevalent in other external anatomical sites considered as follows (Figure 1): *Staphylococcus* spp. was the most common genera found in the ear and the second most common, after *Pseudomonas* spp., in the nose; *Enterococcus* spp. was the most prevalent genera found in the rectum closely followed by *Escherichia* spp. In the oral cavity, *Candida* spp. was the most prevalent genera followed by *Lactobacillus* spp. and *Streptococcus* spp. With a special focus on the thanatomicrobiota, *Enterococcus* spp. was also the most common genera found in the internal organs (Figure 2) and, in particular, in the heart, liver and spleen closely followed by *Candida* spp. and *Pseudomonas* spp. In the brain *Candida* spp. has resulted the most prevalent genera followed by *Enterococcus* spp. and *Pseudomonas* spp. Based on these results, the genera of the bacterial community cultured from swabs of external and internal areas were grouped in their corresponding phyla and distributed according to PMI. 

The isolation frequency of genera for epinecrotic communities and for thanatomicrobiota is shown in Figure 3 and Figure 4, respectively. For the epinecrotic community, the distribution of phyla among the five external anatomical sites (eyes, ears, nose, mouth, and rectum) according to PMI is shown in Figure 3. For the thanatomicrobiota, the distribution of the phyla among the four internal organs (brain, spleen, liver, and heart) according to PMI is depicted in Figure 4. Four main phyla were observed among the epinecrotic bacterial community, as follows (Figure 3): Ascomycota, Actinobacteria, Firmicutes and Proteobacteria.

Distinct compositional changes were observed at the phyletic level throughout decomposition. In the early PMI (<72 h), Proteobacteria was the most common phyla in the eye, ear, nose, and rectum while Firmicutes were prevalent only in the oral cavity. For a PMI > 72 h, Firmicutes were also the most represented bacteria in the eye, ear, oral cavity, and rectum closely followed by Proteobacteria which was the most common phyla in the nose. In the oral cavity, Proteobacteria tended to increase progressively with the PMI, replacing Firmicutes as the dominant phyla, whereas in the eye, Proteobacteria tended to reduce progressively with the PMI, replaced by Firmicutes as the prevalent phyla. For the thanatomicrobiota, only three main phyla were identified and related to the PMI (Figure 4), as follows: Ascomycota, Firmicutes and Proteobacteria. It is worth of mentioning the fact that for the bodies with a PMI < 72 h, all the swabs taken from the internal organs were sterile as no microorganisms were detected using culture-dependent methods. The liver was the first organ to show positive microbiological results in bodies with a PMI range of 72–168 h, suggesting a PM bacterial colonization several days after death, mostly represented by Proteobacteria and Firmicutes. In the other internal organs (brain, heart, and spleen), microbial growth was observed only after 7 days from death. Proteobacteria was the most common phyla in the brain, heart, and spleen, followed soon after by Firmicutes in the heart and spleen and by Saccharomycetaceae in the brain.

## 4. Discussion

Past research studies on the HPM mainly focused on animal models [33,34,35]. They demonstrated successional changes in carrion bacterial communities over decomposition and the potential use of the post-mortem microbial community in PMI estimates [36,37,38]. Compared with previous studies that were based on a high-throughput DNA sequencing approach, this pilot study used MALDI-TOF MS-proteomics profiling based on culture-dependent methods. For estimating the PMI, MALDI-TOF MS has been applied with success in skeletal muscle samples of rats and liver tissue samples, showing very high recognition capability and good cross-validation [39]. Although affected by a lower sensitivity and specificity than metagenomic sequencing, MALDI-TOF has been found to have advantages of cost-effectiveness, rapidity of sample processing and analysis, and greater availability [40]. The aim of our study was to investigate the potential of the MALDI-TOF MS platform to provide information on the epinecrotic and internal bacteria communities according to the PMI and anatomical site. In fact, as with other culture-dependent methods, our study unfortunately suffered from culturing bias, mainly related to the ability of microbes to grow under different culture conditions [41]. However, the results obtained using the MALDI-TOF platform confirmed some previous findings that: (1) the HPM is dominated by bacteria mainly from the Firmicutes and Proteobacteria phyla [9,10,38]; (2) there is a PM microbial community turnover during decomposition that varies according to the PMI and to the depletion of nutrients as putrefaction progresses [17,23]. The HPM became increasingly different from starting communities over time in the internal organs as well as at skin sites, as observed in other reports [9,10]. In previous studies Proteobacteria abundance has been found to increase overall throughout decomposition in animal and human models while Firmicutes and Bacteroidetes decrease [42,43]. Such expected shifts in the structure of the culturable bacterial community over decomposition is also detectable by MALDI-TOF but depends on the anatomical site and insect colonization [19]. In fact, previous research studies already found that Firmicutes dominated carcasses open to insect colonization, whereas carcasses excluded from insects were dominated by Proteobacteria [44,45].

In our study group representing bodies excluded from insect activity, Proteobacteria clearly increased as PMI progressed in the oral cavity and rectum but not in the eye, whereas Firmicutes were the most prevalent phyla in the eye and the ear both in early and late PMIs, but not in the oral cavity. Such findings in the oral cavity are not consistent with a previous study using 454 pyrosequencing in which Firmicutes were found to increase over decomposition, replacing Proteobacteria as the most abundant phyla in oral samples [37]. In this regard, a large-scale survey of 188 death cases representing an urban population documented that most variability over decomposition occurred in the microbial communities of the mouth [17,46]. It was found that anatomic location can influence the microbial diversity, with the rectum and eyes having the most phylogenetic diversity compared with the ears, nose, and mouth having the least diversity [17]. These findings cannot be confirmed by our study mainly because of the lower sensitivity of MALDI-TOF. However, according to Pechal et al [17] and Metcalf et al. [9], it is worth mentioning that external microbial communities are more subject to physical and environmental interactions, whereas internal communities are more influenced by biochemical processes. 

Several factors, intrinsic (i.e., age, sex, infection, and metabolic diseases) and extrinsic (i.e., environment, nutrition, illicit or prescribed drugs, insect activity, and open wounds), can affect the structure of the HPM. Open wounds can be a possible breach for the entry of bacteria, especially if the wounds are located in the vicinity of the sampling sites. Infection and metabolic diseases can influence the internal communities as the widespread presence of bacteria in the bloodstream before death favor the development of the HPM. In our study group only three bodies with a PMI < 120 h showed open injuries to the head in the vicinity of sampling sites (eyes, nose, ear and oral cavity that were not directly injured), and another four victims died due to infection diseases such as pneumonia (three cases with a PMI < 96 h) and septic shock (1 case with a PMI > 168 h). Although these cases could have a confounding role in the interpretation of microbiological results, they were not excluded from the series of bodies because the aim of the study was to detect the overall bacteria dynamics of the epinecrotic community and thanatomicrobiota taking account of all potential affecting factors in bodies of the most common judicial interest, mostly represented by violent deaths (often with large skin defects), as well as by victims who died due to natural causes such as infection diseases.

Our results show a more pronounced diversity of epinecrotic communities (four phyla detected in total) compared with the thanatomicrobiota (only three phyla cultured). A survey of 28 bodies already detected increased richness in the mouth and other mucosal or skin surfaces compared to the internal organs, because microorganisms residing on the surface are more affected by either abiotic (i.e., humidity, temperature, and pH) or biotic factors (i.e., insects and scavenger activities) [47,48,49]. Furthermore, internal sites are usually sterile because they become colonized by PM bacteria only after death [39]. In our study group, all the swabs taken from the internal organs of bodies with a PMI < 72 h were found to be sterile as no microorganisms were detected using the culture-dependent method. This is quite interesting as the internal organs in two out of the three victims who died due to pneumonia were also found to be sterile but with a PMI < 72 h. The liver was the first internal organ to show positive microbiological results in bodies with a PMI > 72 h due to its anatomical location close to pancreatic enzymes, stomach acids, and gallbladder fluids, and reservation of blood coming from the microbe-rich intestinal tract, an excellent growth substrate from which the surrounding tissues can be invaded [11]. Compared with the liver, thanatomicrobiota was detectable in the brain, heart, and spleen only in bodies with a PMI > 168 h, as putrefaction progressed and bacteria colonized internal organs because of the widespread presence of microbes in the bloodstream (especially in the victim who died due to septic shock). This is in agreement with previous studies suggesting that agonal spread or PM translocation of bacteria from mucosal/skin surfaces or from the intestine or other internal organs into the blood are less common than is often assumed [50,51]. Previous studies found that microbial richness increases after death while diversity decreases so that the microbial community tends to become more similar over decomposition [17,39,48]. Our results confirm a more pronounced richness of the PM microbial community in the early PMI compared with a late PMI, especially in the oral cavity and the rectum. Microbial community stability up to two days after death has been demonstrated in a large-scale survey of death cases [17]. Such stability in the early PMI was not really confirmed in our sample. This might be related to the limited cohort study and the period spent in coolers after death as the freezing effect can easily influence the initial structure of the microbiome [37]. In fact, although abundant in human decomposed bodies and capable of translocating from healthy intestines into the blood just a few hours after death, anaerobic bacteria mostly grouped in the Bacteroidetes phylum or in the order of Clostridiales were not found in the present study [11]. They were not detected probably because of the poor sensitivity of the culture-dependent MALDI-TOF, as not all the PM genera were able to grow under the selected culture conditions.

## 5. Strengths and Limitations

The limitations of this pilot study are mainly related to the small sample size, represented by 18 violent and natural deaths, also including victims who died due to infection diseases. However, although several intrinsic and extrinsic factors can affect the structure of the HPM, MALDI-TOF can still detect the overall microbial community turnover of the most prevalent phyla, from external and internal anatomical sites, during decomposition. In this regard MALDI-TOF is a promising method for PMI estimation [19], but no reliable algorithms are yet available based on a statistical correlation between MALDI-TOF results and PMI. The main advantages of the MALDI-TOF are simple sample handling (including a brief cultivation time and low quantity of inoculum required for identification), good reproducibility, and high speed and throughput [52]. The limitations are mainly related to its low sensitivity due to the culture-dependent method and the bias in the identification of new isolates, possible only if the spectral database contains peptide mass fingerprints of the type strains of specific genera and species [24]. Because MALDI-TOF MS is becoming a technique of choice in routine microbiological laboratories, the development of reference databases and user-friendly software for comparisons and analyses would further increase the application of this technique in the forensic context. 

## 6. Conclusions

According to similar studies on microbial indicators and PMI estimates, microbes can be used as predictors but the surrounding environment and confounding factors need to be taken into account [32]. The authors are aware that the results of this pilot study cannot have any practical application in the forensic field without cross-validation by DNA sequencing and further research in the scientific community. In order to improve MALDI-TOF results in forensic practice, it is mandatory to avoid contamination during the storage of bodies in the coolers and at the autopsy by using sterilized instruments. In the near future, we intend to increase the series of bodies with known PMI, differentiated between violent and natural deaths, to be sampled at more regular intervals of hours. The preliminary results of this pilot study could be the starting point for future research studies or, at least, could be useful for comparison with other similar experimental studies using MALDI-TOF MS.

## Figures and Tables

**Table 1:** The study group represented by 18 bodies with details of each case regarding sex, age, death scene, PMI, stage of decay, manner, and cause of death. Legend: y: years; m: months; M: male; F: female; h: hours; PMI: post-mortem interval.

Case #	Age/Sex	Death Scene	PMI (h)	Season	Stage of Decay	Cause of Death	Manner of Death
1	53 y/M	Indoor (Home)	24–36	Winter	Fresh	Hanging	Suicide
2	61 y/M	Indoor (Home)	96–120	Winter	Fresh with early signs of decomposition	Blunt injuries to the head	Homicide
3	38 y/M	Outdoor	36–48	Winter	Fresh	Fatal fall from great height	Suicide
4	57 y/M	Indoor (Hospital)	72–96	Spring	Fresh with early signs of decomposition	Respiratory failure due to pneumonia	Natural
5	40 y/M	Indoor (Hospital)	288–312	Winter	Fresh/Bloated	Multi Organ Failure	Natural
6	45 y/F	Indoor (Hospital)	216–240	Winter	Fresh/Bloated	Septic shock	Natural
7	45 y/M	Indoor (Hospital)	72–96	Spring	Fresh	Gunshot injuries to the head	Homicide
8	20 y/M	Indoor (Hospital)	72–96	Spring	Fresh	Blunt injuries to the head	Homicide
9	80 y/M	Indoor (Hospital)	36–48	Spring	Fresh	Blunt Trauma	Road Accident
10	6 m/M	Indoor (Home)	24–36	Spring	Fresh	Respiratory failure due to pneumonia	Natural
11	51 y/M	Indoor (Prison)	96–120	Spring	Fresh with early signs of decomposition	Subarachnoid hemorrhage	Natural
12	82 y/M	Indoor (Hospital)	336–360	Summer	Fresh/Bloated	Fatal fall from great height	Suicide
13	4 m/M	Indoor (Home)	24–36	Summer	Fresh	Respiratory failure due to pneumonia	Natural
14	41 y/M	Indoor (Hospital)	168–192	Summer	Fresh/Bloated	Liver failure in cirrhosis	Natural
15	20 y/M	Indoor (Hospital)	96–120	Winter	Fresh with early signs of decomposition	Blunt Trauma	Road accident
16	53 y/M	Outdoor	240–264	Spring	Bloated	Myocardial infarction due to coronary artery disease	Natural
17	22 y/M	Indoor (Hospital)	36–48	Spring	Fresh	Blunt Trauma	Road accident
18	70 y/M	Outdoor	36–48	Summer	Fresh	Arrhythmogenic cardiomyopathy	Natural

# meas number of the case.

## Data Availability

The data presented in this study are available on request from the corresponding author.
